# Taxonomy of access to mental healthcare for economically marginalized women during a public health crisis: a qualitative study of obstetric professionals and perinatal women during the COVID-19 pandemic

**DOI:** 10.3389/fpsyt.2026.1783084

**Published:** 2026-07-15

**Authors:** Azure B. Thompson, Ana J. Schaefer, Clevanne Julce, Martha Zimmermann, Leah Ramella, Dienta Rochani, Tiffany A. Moore Simas, Wendy Davis, Nancy Byatt, Thomas I. Mackie

**Affiliations:** 1 State University of New York (SUNY) Downstate Health Sciences University, School of Public Health, Brooklyn, NY, United States; 2 University of Massachusetts Amherst, School of Public Health and Health Sciences, Amherst, MA, United States; 3 Department of Psychiatry and Behavioral Sciences, UMass Chan Medical School, Shrewsbury, MA, United States; 4 University of New England, College of Osteopathic Medicine, Biddeford, ME, United States; 5 Department of Obstetrics & Gynecology, Psychiatry & Behavioral Sciences, Population & Quantitative Health Sciences, University of Massachusetts Chan Medical School, Worcester, MA, United States; 6 Department of Psychiatry and Behavioral Sciences, UMass Chan Medical School, Worcester, MA, United States; 7 Postpartum Support International, Portland, OR, United States

**Keywords:** economically marginalized, mental healthcare, obstetric professionals, perinatal women, public health crises, perinatal period

## Abstract

**Background:**

Mood and anxiety disorders are the most common pregnancy complications in the United States. Global public health crises increase the risk of these disorders, impact obstetric professionals that serve perinatal women, and introduce new challenges to accessing mental healthcare, particularly for economically marginalized perinatal women. Accordingly, the study purpose is to develop a taxonomy of mental healthcare access during public health crises for economically marginalized perinatal women.

**Methods:**

In-depth interviews were conducted with obstetric professionals (n=14) serving economically marginalized perinatal women during the COVID-19 pandemic. Our research team employed a modified grounded theory to develop a taxonomy comprising dimensions, components and factors for perinatal mental healthcare access. Results from obstetric professionals were then triangulated with data from economically marginalized perinatal women (n=24) who attempted to access mental healthcare during the COVID-19 pandemic.

**Results:**

Obstetric professionals enumerated factors related to a six dimension taxonomy of access, including approachability, availability, affordability, accessibility, accommodation, and acceptability. Dimensions were triangulated and found to align with experiences of perinatal women. Each dimension included multiple components and factors influential to the respective component. For example, the dimension of affordability included direct mental healthcare costs (the component) which reportedly increased for patients due to a rise in un- and under-employment and loss of employer-sponsored health insurance (the factors).

**Conclusion:**

Findings highlight opportunities for policymakers and healthcare professionals to identify components impacted across the six dimensions of access for economically marginalized perinatal women and respond to the factors influential. Interventions could include ongoing support to normalize mental healthcare, collaborations with community-based organizations, prioritization of patient-centered and collaborative care models.

## Introduction

More than one in five women who are pregnant or within one year postpartum experience a mood or anxiety disorder (1, 2). These disorders are a pressing public health issue particularly among perinatal women because they are associated with increased risks of suicide, drug overdose, and severe maternal health complications (3–6), in addition to preterm birth, low birthweight (7), delayed language development and behavioral disorders (4). Public health crises further intensify these challenges. Not only have rates of mood and anxiety disorders increased in the last two decades (8), perinatal mental health has worsened with the onset of the COVID-19 pandemic (9, 10). For example, in a systematic review of perinatal mental health outcomes during the COVID-19 pandemic, rates of clinically significant depression increased to one in four (26.7%) pregnant and one in three (32.7%) postpartum women (1, 2, 10).

During the COVID-19 pandemic, perinatal women faced heightened uncertainty regarding risks to their own health and that of their infants, as well as increased emotional distress related to social isolation compared with non-perinatal women (10–12). In addition, perinatal women experienced disruptions in prenatal care, which can affect emotional and mental health (10, 13, 14). In one study, more than 25% of pregnant women canceled in-person prenatal visits during the COVID-19 pandemic (13). These types of disruptions in care are associated with an increase in anxiety and depression symptoms (10), symptoms that are experienced among perinatal women with greater prenatal care needs, such as those experiencing high-risk pregnancies (10).

Perinatal women also faced additional barriers to accessing mental healthcare during the COVID-19 pandemic (15, 16). These barriers are particularly pronounced for women who are economically marginalized such as those insured through Medicaid, uninsured, or low-income (17). Routine and effective screening for perinatal mental health disorders is often the first barrier to care (18). Perinatal women report not sharing mental health symptoms with their healthcare professional for a variety of reasons that include not believing that they need mental healthcare services (19) or fearing that reporting symptoms will result in child protective service involvement and loss of custody (19). If a perinatal individual is identified as needing mental health care, accessing the needed care is challenging due to the shortages of mental health professionals (20). Among mental health professionals who served perinatal women, 42% reported that their patients had to wait more than two months for a mental health appointment (18).

The COVID-19 pandemic affected the obstetric workforce, inclusive of obstetrician/gynecologists, licensed midwives, nurse practitioners, and other licensed obstetric care practitioners, in various ways (21), in ways that may influence access to mental health care. During the pandemic, workflow and care management models changed rapidly to adhere with COVID-19 risk mitigation strategies (22–24). Simultaneously, the obstetric workforce reported increased levels of stress and burnout (25). These staff shortages and shifts in care referral pathways directly affected access to and delivery of both obstetric and mental healthcare (23, 24, 26).

During these challenges, recent initiatives have increasingly positioned obstetric professionals and settings at the forefront of perinatal mental healthcare access. In 2023, the American College of Obstetricians and Gynecologists (ACOG) issued recommendations stating that obstetric professionals should screen for depression and anxiety using standardized, validated measures (27). Obstetric settings have increasingly been recognized by policymakers and clinicians as ideally suited to address perinatal mental health because obstetric professionals are in routine contact with perinatal women. For example, the Alliance for Innovation on Maternal Health (AIM) also issued a patient safety bundle for perinatal mental health conditions outlining structured approaches to integrate relevant evidence-based practices into obstetric settings.

Yet the feasibility of routine screening in obstetric settings are likely to be markedly impacted by public health crises, such as the COVID-19 pandemic. Rapid changes in healthcare delivery, coupled with the disproportionate impact of such crises on under-resourced obstetric settings and economically marginalized perinatal women, can create substantial barriers to consistent screening and referral practices. The role of obstetric professionals in delivering mental healthcare is well documented (18, 28, 29). However, the factors that influence the ability of obstetric professionals to facilitate mental healthcare delivery to economically marginalized perinatal women amidst public health crises remains relatively unknown (15, 18, 28, 30).

The purpose of this qualitative study is to develop a taxonomy of perinatal mental healthcare access among economically marginalized perinatal women during a public health crisis. Our goal is not only to understand the experiences of access to care, but also to generate findings that can inform preventive and responsive policies and practices for economically marginalized populations within obstetric and perinatal care settings. Using qualitative methods, we explore both barriers and facilitators to perinatal mental healthcare when healthcare systems are performing during crisis, specifically the COVID-19 pandemic. We then identify dimensions of access from those factors identified.

To ensure that our findings are actionable and easily applied, we will develop a taxonomy guided by grounded theory analysis. The benefit of a taxonomy it is a structured system for organizing information into categories based on shared characteristics or relationships. Through the development of this structed system, we will provide a logical framework that shows how concepts relate to one another, hierarchically. Through this approach, the resulting taxonomy will provide a clear, organized framework that captures the influences on access to perinatal mental healthcare among economically marginalized perinatal women during times of public health crisis and offers a practical tool to guide future policy.

To conceptualize access, we use 6 A’s of Access that builds on the five dimensions identified by Penchansky & Thomas, 1981 (31)—availability, accessibility, accommodation, affordability, and acceptability—while incorporating a sixth dimension, approachability, as introduced by Levesque et al., 2013 (32). The original 5 A’s framework emphasizes the “fit” between patient needs and the capacity of the healthcare system. However, scholars have noted that it does not fully account for a patient’s ability to initially recognize a need for care and seek services.

Approachability addresses this gap and is especially important for marginalized populations, who may face barriers to considering care due to factors such as fear of consequences of reporting symptoms (19). By applying this expanded 5 A’s framework, this study aims to identify specific gaps across dimensions of perinatal mental health access that emerged during the COVID-19 pandemic.

## Methods

### Study design

Consistent with the role of qualitative inquiry in research (33), we conducted semi-structured, in-depth interviews to explore an understudied area, specifically the factors for mental healthcare access for women economically marginalized during a global public health crisis. The global health crisis during which the study was conducted was the COVID-19 pandemic, which was declared a global pandemic from March 2020 through January 2023 (34, 35). The study was conducted between October 2021-January 2022.

Our qualitative research methods team was interdisciplinary and consisted of medical and public health practitioners in addition to researchers and medical sociologists. We first explored the dimensions of access as reported by obstetric professionals in the United States and then factors influential to access as a multi-dimensional construct. Systematic reviews on perinatal mental healthcare access make calls for triangulation of understandings across impacted community partners, including patients and obstetric professionals (36). Therefore, our team then triangulated these data by investigating whether results from the interviews conducted with perinatal women who are economically marginalized and with lived experiences of accessing care for perinatal mood and anxiety disorders during the COVID-19 pandemic, themselves, converged with experiences of obstetric professionals who served this population during this time.

We engaged triangulation through an exploration of the extent to which data from perinatal women corroborated findings from obstetric professionals (37). Accordingly, interviews were conducted with both (1) obstetric professionals who served perinatal women who were economically marginalized, and (2) economically marginalized perinatal women who sought mental healthcare during the pandemic.

### Sample

#### Obstetric professionals

The obstetric professionals included obstetricians, nurse practitioners, physician assistants and certified nurse mid-wives who provided perinatal care during the COVID-19 pandemic. Our team purposively sampled these obstetric professionals as ambulatory obstetric practices are integral sites of perinatal mental health screening and assessment; for example, the recommended standard of perinatal care in obstetric settings is universal screening for perinatal depression and anxiety (27, 38). To identify a national sample of obstetric professionals, social media posts, including information about the study and a link to the eligibility survey, were distributed through Postpartum Support International, an international organization that aims to promote awareness, prevention, and treatment of perinatal mental health issues, and the American College of Obstetricians and Gynecologists (ACOG). Sixty-eight perinatal care professionals responded of which 38 were eligible or not contacted. Of those not eligible or not contacted, participants were either (1) not part of the perinatal care professional workforce (n=12), (2) declined to be interviewed or did not answer the question to opt into the interview (n=4), (3) did not meet criteria for socioeconomic situation of patients served (n=5), or were not contacted as recruitment was complete (n=9). Of the remaining participants, 27 were obstetric professionals (i.e., obstetricians, nurse practitioners, physician assistants and certified nurse mid-wives). Given our focus on experiences in obstetric healthcare settings, doulas (n=11) were not included in the present analysis. Of the remaining 27 eligible obstetric professionals, 9 could not be contacted after up to five attempts, and one was not able to participate during the study recruitment time period. Seventeen obstetric professionals were able to be contacted and consented. Three obstetric professionals were lost to follow-up after consent, resulting in a total sample of 14 obstetric professionals participating in the semi-structured qualitative interviews. Surveys and semi-structured in-depth interviews were conducted via telephone or video conferencing platforms (e.g., Zoom) and lasted for approximately 60 minutes.

For data presented in this paper, obstetric professionals were eligible if they: (1) were 18 years of age or older, (2) could read, understand, and speak English, (3) had access to a telephone to complete the interview, (4) had access to the internet to complete enrollment, and (5) provided perinatal care as an obstetrician, nurse practitioner, physician assistant or certified nurse mid-wife since March 15, 2020, (6) reported providing care to patients who were insured through Medicaid or public insurance or (7) reported working in a practice serving women in a challenging socioeconomic situation.

#### Perinatal women

To facilitate a national sample of perinatal women, Postpartum Support International, a large community-based organization, distributed recruitment materials via its social media outlets on Facebook, Instagram, and Twitter. The flyer included general information about the study and a link to a REDCap eligibility screening survey (39, 40). Women were eligible if they: (1) were 18 years of age or older, (2) could read, understand, and speak English or Spanish, (3) had access to a telephone to complete the interview, (4) had access to the internet to complete enrollment, (5) reported receiving prenatal care since March 15, 2020 or delivered a baby since May 15, 2020, (6) reported having sought access to mental healthcare from March 15, 2020 until February 21, 2022, (7) selected at least one indicator of low-income status (e.g. health insurance status, food insecurity, housing insecurity, participation in Special Supplemental Nutrition Program for Women, Infants, and Children (WIC) or Early Head Start), and 8) answered “yes,” to the question “Did you have concerns about your emotional or mental health since March 15, 2020?” If women were eligible, they were able to enter their contact information.

A stratified sampling approach was used to identify the sample interviewed. We routinely reviewed the sample and purposively sampled from the eligibility screening survey to ensure the racial, ethnic, and language diversity of our sample. Surveys were administered to perinatal women prior to completing the semi-structured in-depth interview. Interviews with perinatal women were then conducted in English or Spanish in an approximately 60-minute interview by phone or video conferencing.

### Measures development

Brief surveys and in-depth semi-structed interview guides for obstetric professionals and perinatal women were developed (Appendix 1-2). Surveys queried relevant sociodemographic information and interview guides explored comparable domains, including: (1) access to and experiences of quality perinatal healthcare amidst the pandemic, (2) access to and experiences of perinatal mental healthcare amidst the pandemic, and (3) the experiences of and perceived impact of delivery system transformation amidst the pandemic (e.g., telehealth, mask mandates etc.).

Surveys and semi-structured interview guides for both samples were developed by the interdisciplinary research team in consultation with extant literature (18, 41) and a process of iterative review with three State Advisory Councils and a National Council (Appendix 3), as described in the section, entitled “community engagement approach” below. The survey and interview guide were also piloted with two women who did not participate in the study, itself.

#### Obstetric professional survey and interview guide

The survey for perinatal professionals included 51 questions, about the following: (1) gender, race, and ethnicity, (2) professional experience, (3) practice type, and (4) perinatal mental health screening and treatment practices. The semi-structured interview guide included 40 questions with subsequent probes. For this sample, we explored topics by querying the experiences of the obstetric professionals amidst the COVID-19 pandemic. We asked respondents to speak to their experiences during the pandemic and to make comparisons, as appropriate, to their experiences in providing care before the pandemic. The present paper leverages questions asked on the experiences in providing perinatal care and facilitating the delivery of mental healthcare amidst the pandemic, specifically for economically marginalized populations served. The obstetric professional survey and interview guide are in **Appendix 1** and **Appendix 2**, respectively.

#### Perinatal individual survey and interview guide

The survey fielded to perinatal women included 68 questions that queried: (1) gender, race, and ethnicity, (2) indicators of low-income, (3) location where obstetric care was received, and (4) current mental health symptoms including depression as assessed by the Edinburgh Postnatal Depression Scale (EPDS), Generalized Anxiety Disorder-7 (GAD-7), and Primary Care Post Traumatic Stress Disorder Screen for DSM-5 (PC-PTSD-5). The semi-structured interview guide for perinatal women included 39 questions with subsequent related probes. We queried topics articulated above by asking about their personal experiences in seeking perinatal mental healthcare amidst the COVID-19 pandemic. The perinatal women survey is in **Appendix 3** and interview guide in **Appendix 4**.

### Study procedures

#### Perinatal women

Research team members also conducted semi-structured in-depth interviews with perinatal individuals between November 08, 2021, and February 28, 2022. Perinatal individuals were offered in the interview in English and Spanish. Perinatal individuals received a $40 gift card for renumeration. Interviews continued until saturation of themes was achieved on factors influential to mental healthcare access, such that “no additional data are being found whereby the [researcher] can develop properties of the category” (35).

#### Obstetric professionals

Members of the research team conducted the interviews. Obstetric professionals were offered the interview in English as Spanish-speaking obstetric professionals were anticipated to be bilingual. Following the interview, obstetric professionals were offered a $40 gift card for remuneration. Interviews were conducted until saturation of themes described above was achieved, such that “no additional data are being found whereby the [researcher] can develop properties of the category” (42). Consistent with the formulation of Glaser and Strauss (1967), we achieved thematic saturation when our theoretic model on dimensions of access stabilized.

### Community engagement approach

We convened four Advisory Boards. Participants were community members who represent diverse populations that worked directly with the research team to facilitate community input by providing feedback and advice on all aspects of the research process and as such provided a voice to the concerns and interests of their respective communities. The study was part of a three-state comparative effectiveness study that included Advisory Boards from each state (Massachusetts, New Jersey, Washington) and a National Advisory Board. The National Advisory Board comprised 13 representatives from national advocacy and service organizations, professional societies, and the research community. The Massachusetts Advisory Board included seven representatives, the New Jersey Board included 13 and Washington included 10. Although the specific composition of each Board varied by state, the State Advisory Boards were composed of at least one representative from each of the following affiliations: (1) individual with lived experience of perinatal depression, (2) perinatal healthcare professional, (3) relevant community-based programs, (4) health plan, or (5) a relevant state policymaker whether from the Department of Health and Human Services, Mental Health, Maternal and Child Health, or Medicaid agency.

Our research team employed strategies to facilitate and support the meaningful engagement of research partners including Advisory Board members in informing the project methods, structures, processes, and continuous quality improvement measures and approaches (citation withheld to preserve author anonymity). Over a series of quarterly meetings, the Advisory Boards informed four areas of the current research study. First, the Boards convened at the outset of the project to prioritize the research topic and proposed question, specifically the focus on perinatal mental healthcare access for the economically marginalized amidst a public health crisis. Second, the Boards were convened to inform the sampling framework and recruitment procedures, including the range of obstetric professionals included and our collaboration with Postpartum Support International to facilitate national. Third, the Boards informed the study methods and measures, including both interview design and content. The Boards reviewed the domains to be covered in both the survey and interview guides for both obstetric professionals and perinatal women. Members of the Board suggested beginning the guide for perinatal women with questions asking generally about experiences receiving perinatal healthcare before inquiring on experiences accessing mental healthcare specifically. Additional feedback informed how we inquired on the experiences across the perinatal mental healthcare pathway (i.e., detect, assess/diagnose, triage, treat, follow-up). Finally, the results presented in this paper were provided to each of the Boards to facilitate interpretation of study findings, contributing to this article’s discussion and policy and programmatic implications.

### Data analysis

We used a grounded theory approach (43) to analyze data from obstetric professionals (n=14). First, interview recordings were transcribed. Transcripts were then checked for accuracy and de-identified. Second, the research team familiarized themselves with the data, listening to recordings and reading transcripts. Third, the research team reviewed transcripts employing both deductive (based on extant literature, research team and Advisory Board members’ insights), and inductive (grounded in the data itself or “open coding”) code development. Finally, two or more trained investigators performed line-by-line coding of each transcript using the developed codebook. Investigators then reconciled the respective coding against each other and arrived at consensus on the codes line-by-line. The coded data was then entered into Dedoose, a qualitative software program used to manage, organize data, and support the analysis of data (44).

Following initial coding of all transcripts, the research team conducted axial and selective coding. During axial coding, we examined relationships among codes and grouped them into categories based on conceptual connections. The team reviewed coded data from obstetric professionals and collaboratively discussed categories identified and their interrelationships.

Selective coding, the final stage of grounded theory analysis, involved integrating and refining categories developed during axial coding into a coherent explanatory framework. Through this process, we identified categories that described distinct dimensions of access to perinatal mental healthcare. As these categories were further refined, we observed substantial alignment with Penchansky and Thomas’s (1981) 5 A’s of Access”: availability, affordability, accessibility, accommodation, and acceptability (31).

To further examine these relationships, we returned to the data and systematically reviewed the categories in relation to the 5 A’s framework. This process allowed us to validate, refine, and consolidate the dimensions of access identified in the data and to clarify how access to perinatal mental healthcare was experienced and enacted.

As the analysis progressed, we recognized that some aspects of the access process extended beyond the original 5 A’s framework. Consequently, we drew on the expanded access framework proposed by Levesque et al. (2013) (32), which incorporates approachability—the extent to which individuals can recognize their need for care and the extent to which healthcare professionals facilitate engagement with services. Integrating this additional dimension enabled us to develop a more comprehensive taxonomy of access and an explanatory framework that more fully captured the pathway to perinatal mental healthcare.

**Figure 1:**
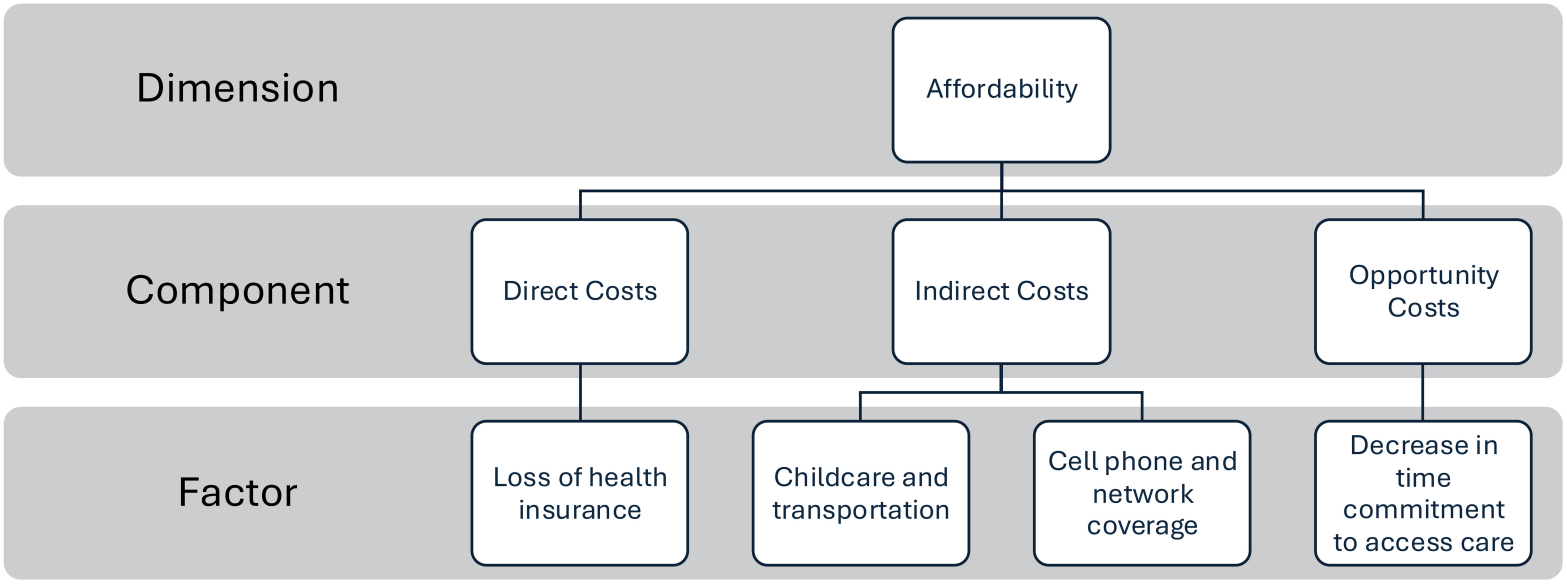
Illustrative examples of hierarchical coding structure employed in identifying dimensions, components, and influential of the respective component.

Our team then coded the distinct component within the respective dimensions of access. We found that unique factors (i.e., barriers, facilitators) influenced each component of the respective dimension and therefore aligned the factors to the respective component. As illustrated in **Figure 1**, this resulted in our coding of the dimension, the respective components and then factors that influenced the respective components. For example, our analyses identified the dimension of affordability, with one *component* of affordability being the direct costs to patients associated with out-of-pocket expenditures for mental healthcare and medications. A reported *factor* that influenced the impact of the direct costs of perinatal mental healthcare on accessing perinatal mental healthcare was the rise in unemployment among patients and the resulting loss of employer-sponsored health insurance amidst the COVID-19 pandemic. (See **Figure 1**).

After completing the identification of dimensions identified from obstetric professional data, we triangulated findings with data from perinatal women. The triangulation approach used was convergence, defined as comparing data from different sources to corroborate findings (39). Specifically, we applied the six dimensions of access, identified in the obstetric professional analyses, to investigate whether findings corroborated with perspectives provided by perinatal women. To do this, we analyzed transcripts from the perinatal women using categories developed for the 6 dimensions of Access, as articulated by the obstetric professionals. We then reviewed and coded all the transcripts of perinatal women to identify convergence or divergence of perspectives on the dimensions of perinatal mental healthcare access derived from obstetric professionals. We represent the results of these efforts below, providing the operational definition for each dimension and then the component and illustrative examples and associated factors underneath each.

## Results

Fourteen obstetric professionals completed a survey and a semi-structured interview. The majority (12, 86%) identified as female. Thirteen (93%) participants identified their race as white with one participant providing no response. Six respondents (43%) identified as obstetricians or gynecologists, four (29%) identified as nurse practitioners or physician assistants, and four (29%) identified as midwives. Complete sample details are provided in **Table 1**.

**Table 1:** Sociodemographic Characteristics of Obstetric Professionals and Perinatal Women with Experience of Emotional or Mental Health Concerns.

	Obstetric Professionals	Perinatal Women with Experience of Emotional or Mental Health Concerns
**Total (n)**	**14**	**24**
Sex, n (%)^a^		
Female	12 (85.7)	24 (100)
Male	1 (7.1)	0 (0)
No response	1 (7.1)	0 (0)
Race, n (%)		
Black / African / African American	0 (0)	7 (29.2)
White/Caucasian	13 (92.9)	12 (50.0)
Other	0 (0)	5 (20.8)
No response	1 (7.1)	0 (0)
Ethnicity, n (%)		
Hispanic or Latino	0 (0)	9 (37.5)
Non-Hispanic or non-Latino	12 (85.7)	15 (62.5)
Other	1 (7.1)	0 (0)
No response	1 (7.1)	0 (0)
Professional title, n (%)^b^		
Ob/Gyn or Attending Physician	6 (42.9)	N/A
Nurse Practitioner/Physician Assistant	4 (28.6)	N/A
Midwife	4 (28.6)	N/A
**Years at current position, average (STD)**	14.25 (10.8)^c^	N/A
Location of practice, n (%)		
Rural community	1 (7.1)	N/A
Suburban community	3 (21.4)	N/A
Urban community	9 (64.3)	N/A
No response	1 (7.1)	N/A
Practice type, n (%)		
Private or community practice	1^d^ (7.1)	N/A
Community health center	3^e^ (21.4)	N/A
Academic medical center	10 (71.4)	N/A
Health Insurance, n (%)		
Private	N/A	14 (58.3)
Public	N/A	8 (33.3)
Other	N/A	2 (8.3)
No insurance	N/A	1 (4.2)
Financial questions, n (%)		
WIC enrollment^f^	N/A	14 (58.3)
Headstart or early Headstart enrollment	N/A	4 (16.7)
Food insecurity	N/A	12 (50.0)
Utility bill insecurity	N/A	12 (50.0)
Housing insecurity	N/A	6 (25.0)
Current employment status, n (%)		
Unemployed and looking for work	N/A	6 (25.0)
Unemployed and not looking for work	N/A	3 (12.5)
Part time or temporary work	N/A	2 (8.3)
Full time work	N/A	12 (50.0)
No response	N/A	2 (8.3)
Mental Health Screening, mean (SD)		
EPDS^g^ Score, mean (SD)	N/A	11.2 (7.2)
GAD-7^h^ Score, mean (SD)	N/A	6.65 (4.82)
PC-PTSD-5^i^, mean (SD)	N/A	2.38 (1.80)

a. Respondents were offered several options for response for each category, we have only represented categories in which respondents had indicated a response.

b. Numbers may add to more than 100% as respondents could select more than one affiliation.

c. Three respondents did not indicate length of time in current position.

d. Numbers may add to more than 100% as respondents could select more than one practice type as necessary.

e. Numbers may add to more than 100% as respondents could select more than one practice type as necessary.

f. Special Supplemental Nutrition Program for Women, Infants, and Children (WIC)

g. Edinburgh Postnatal Depression Scale (EPDS)

h. Generalized Anxiety Disorder (GAD-7)

i. PC-PTSD-5 Primary Care PTSD Screen for *DSM-5* (PC-PTSD-5)

### Dimensions of access

**Figure 2:**
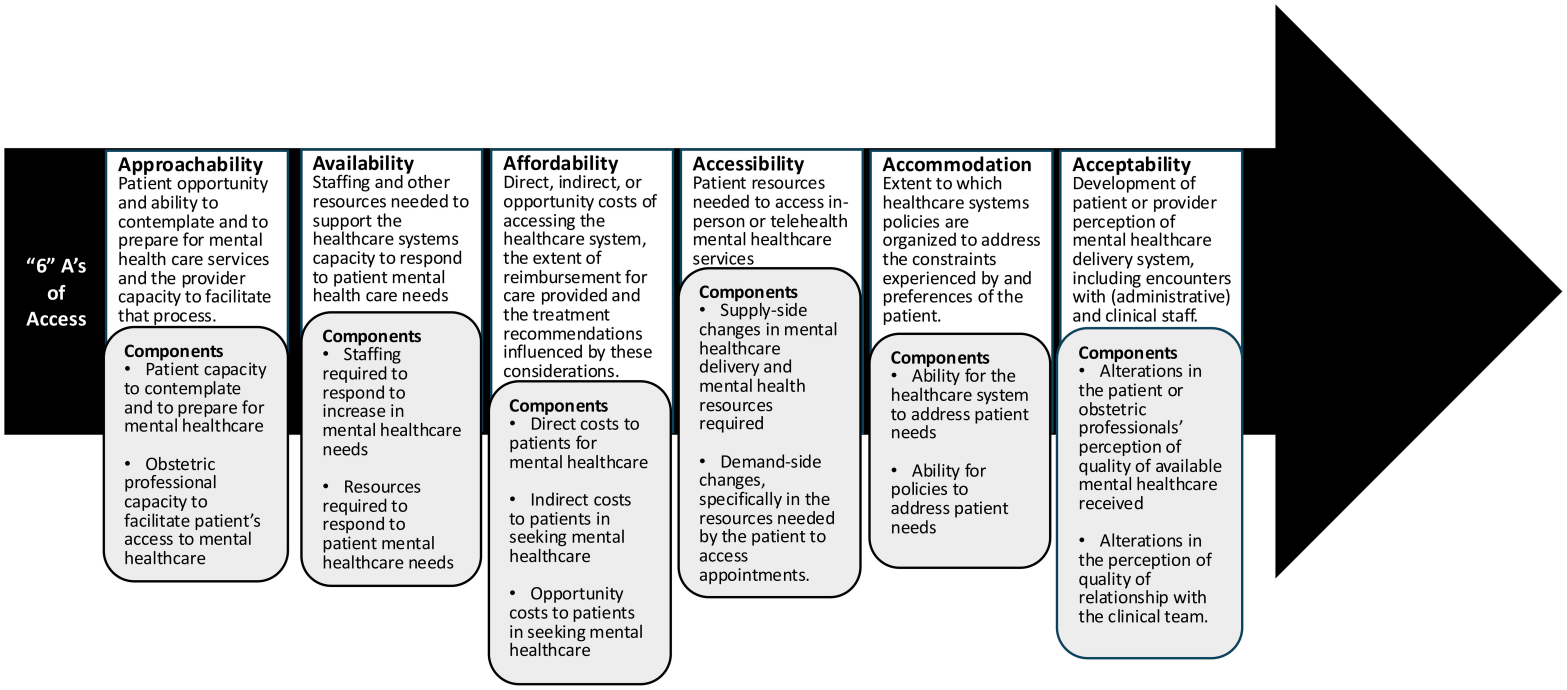
Dimensions of access and components of each dimension.

Our analysis produced a six-dimensional taxonomy of perinatal mental healthcare access consisting of approachability, availability, affordability, accessibility, accommodation, and acceptability. Subsequent triangulation with perinatal women demonstrated convergence with each dimension. **Figure 2** illustrates how accessing mental health care generally requires movement from the outermost dimension of access, “affordability,” to the innermost dimension, “acceptability.” The arrow across dimensions of access also intends to indicate that receipt of needed mental healthcare may be impeded by anyone (or combination) of the 6 dimensions of access. In reporting on each dimension of access, we offer the operational definition, an illustrative example, and the components of each dimension of access in turn. **Table 2** then summarizes these findings and provides additional illustrative quotes to substantiate the reported themes.

**Table 2:** Dimensions of Access to Perinatal Mental Healthcare.

Dimension Component(s)	Triangulation of the components of 6 A's of Access by Perinatal Women	
	Obstetric Professionals	Perinatal Women
2.A. Approachability Patient opportunity and ability to contemplate and prepare for mental healthcare and the obstetric professionals' capacity to facilitate that process.		
2.A.1 Patient capacity to contemplate and to prepare for mental healthcare	“Maybe they have, feel more permission to speak up because it's, you know, socially acceptable to be, you know, struggling during a pandemic.” OP6	“To be honest, I was afraid of seeking help because last time, when I asked for help, well I was hospitalized in psychiatry, and I don't want that.” Perinatal Woman (PI) 4
2.A.2 Obstetric professionals' capacity to facilitate patient's access to mental healthcare	“I have to say I feel like we were so into these efforts before the pandemic that it didn't. It didn't feel like we did much different while the pandemic was happening - makes sense.” OP6	“All I can say is that I think that they're [obstetric professionals] so backed up with how many appointments they have and how fast they were trying to get people in and out due to, like, the COVID-19 restrictions that it [Mental health] just wasn't a priority.” PI6
2.B Availability Staffing and other resources needed to support the healthcare system capacity to respond to patient mental healthcare needs.		
2.B.1 Staffing required to respond to increase in mental healthcare needs	“It's gotten incredibly hard to get people into therapy. Supply and demand is just – there's more demand than supply. And so that is true for primary care-based services as well as mental health – based services and counseling services. It's really, um, you know, these people are flooded with folks and there's just not enough providers around.” OP6	“It was incredibly hard to find anyone just because most people [mental health providers] had, like, either shut down their practices or like, were not accepting new patients.” PI2
2.B.2. Resources required to respond to patient mental healthcare needs such as the infrastructure	“Um, I feel like, um, certain things are harder though because there are many more demands on our clinician's time, so we're less available and so I feel like patients are, are waiting longer to, to get care. But telehealth certainly has broken down a lot of barriers.” OP1	We do have several parenting organizations that do that, but yeah, those weren't available the way that - they would've been outside with the pandemic. PI7
2.C Affordability		
Direct, indirect, or opportunity costs of accessing the healthcare system, the extent of reimbursement for care provided and the services sought, and treatment recommendations influenced by these considerations.		
2.C.1 Direct costs to patients for mental healthcare	“Sometimes the medications, I know that another medication may be of a benefit, but they can't afford it.” OP5	“I delayed getting help for several months eventually when I should've gotten it a lot earlier and then didn't go as frequently as I probably should've because we couldn't afford, you know, the sessions were like $ 130.” PI5
2.C.2 Indirect costs to patients for mental healthcare	“In the heart of the pandemic we saw the shift from in person care to offering more virtual and telehealth visits, which seemed to fit the needs of a lot of families that we had been who don't have access to transportation or, you know, just not as consistent access to transportation, those that are lower income who can't afford to be, you know, driving from their home, sometimes more than an hour away, seek care, the type of care that they were looking for.” OP8	Before the pandemic happened, I had to go in person and that's about an hour and a half or hour and 45 minutes, including travel and the [unclear] that I had to be out of the home, but because of the pandemic…all I had to do was hop on the phone for an hour and then I'm off the phone. PI1
2.C.3 Opportunity costs to patients for mental healthcare	“I mentioned it a little bit before, but if they have a lot of children at home and they can't afford childcare and they can't afford to not be at home and they can't go to their appointments. We were also restricting visitors so they couldn't bring their kids with them to their appointments, like they had been able to in the past. And I think that really caused people not to come to their schedule's appointments.” OP4	“I was able to find a counselor who did everything in the house. I'm still able to care for my children if I need to and that's been very beneficial.” PI2
2.D Accessibility Patient resources needed to access in-person or telehealth mental healthcare.		
2.D.1 Supply-side changes in mental healthcare delivery and mental health resources required	“Once, once they get a relationship with a therapist, it's been easy because of telehealth you know, transportation getting there is not as big a deal.” OP3	“And the fact that she had switched to tele-health did help as well since I had a newborn. So, I don't know if she would have done that without COVID or if she still would have just been in person because it did make it easier for me to see her via telehealth.” PI3
2.D.2 Demand-side changes, specifically in the resources needed by patients to access appointments	“A lot of my patients don't have their own transportation, so it was not wanting to come in because they'd have to take the bus and be exposed that way. And then not having money to, say, take an Uber if they wanted more private transportation or not being able to come to appointments for, you know, the policy of no, you know, people with you.” OP2	“She gave me the therapy sessions through Zoom, but at the time, the kids wouldn't go to school or anything and I was living in a small room where I had to be with the kids; it was just the room and the kitchen. So, I couldn't take the therapy sessions with the kids there.” PI4
2.E Accommodation Extent to which healthcare systems and policies are organized in ways to address the constraints experienced by and needs of the patient.		
2.E.1 Ability for the healthcare system to address patient needs	“We were also restricting visitors so they couldn't bring their kids with them to their appointments, like they had been able to in the past. And I think that really caused people not to come to their schedule's appointments.” OP4	“But also my dream is always to have a home birth, so we took as an opportunity to be able to have it now, especially like I'm a postpartum doula, so I've always wanted to and I've been learning more about black midwifery and I've wanted to, I wanted to like give my money, a black midwife and have that experience” PI1
2.E.2 Ability for policies to address patient needs	“So we, we saw that we were creating these barriers for people who needed specific support resources because we were following CDC recommendations and, you know, following the latest guidelines. And then also we saw a lot of, um, as far as barriers go to healthcare, we saw a lot of practitioners turning people away based on their ability to wear proper PPE, their vaccination status, their will and desire for increasing their volume.” OP8	Interviewer: And how did you attend your pregnancy care appointments during the pandemic? Interviewee: Oh, it was very stressful. I was in the unknown and I wore masks and I went by myself and thankfully this was my second pregnancy, so I can't imagine for the ones who this is their first time delivery, but it was very stressful for sure. PI8
2.F Acceptability Development of patient or obstetric professional perception of the receipt of mental healthcare, including encounters with (administrative) and clinical staff.		
2.F.1 Patient or obstetric professionals' perception of quality of available mental healthcare received	“I don't always know how to fix that other than just trying to, like really when I do have them physically in the room with me, trying to make sure I can take care of as many of their needs as possible at that moment, because I do worry sometimes when there's that language barrier, um, and not having kind of that ease of virtual access to people that once they step out of the room, you know, I worry that I, they won't be able to reach me with concerns or I won't be able to reach them to follow up on things.” OP9	“I don't think that they did, like, the EPDS or PHQ9… it was a really short checkup, the, um, the one that I had following up with them and I don't think that they really talked about anything, mental health, it was more like physical.” PI2
2.F.2 Alterations in perception of quality of relationship with clinical team.	“I think - so I - you never feel connected with somebody when you're - you meet them for the first time over the phone or through a video. So I think that has - and yeah, I know I mentioned that before, but that really has affected it. And then I don't think people are very honest when it comes to like phone calls. A little bit better with video calls, but it's not - you can't really get their, like, the full picture of, like, their body language through those platforms. So the face - to - face visits, I think you get a better picture of, of their health and how they're doing mentally.” OP4	“Everything was like very efficient and kind of speeded up. If I had questions, I didn't want to drag out the visit by asking anything. I think I only saw him without his mask once, so I'm not sure I would recognize him without it, and he wouldn't recognize me either. So, effective arm's length.” PI1

### Factors influential across dimensions of access

The taxonomy consisted of dimensions, components, and influential factors. **Table 3** aligns with the text provided below, listing the components for each dimension of access, the factors influential to the respective component, and an illustrative quote for each of the factors identified.

**Table 3:** Dimensions of Access to Perinatal Mental Healthcare, Factors Influencing Change Amidst the COVID-19 Pandemic, and Illustrative Quotes.

Dimension Component(s)	Factor(s)	Obstetric Professional Illustrative Quote
3.A. Approachability Patient opportunity and ability to contemplate and prepare for mental healthcare and the obstetric professionals' capacity to facilitate that process.		
3.A.1 Patient capacity to contemplate and to prepare for mental healthcare	3.A.1i. Openness during the pandemic to share with others preexisting and pandemic related issues with mental health.	“Previously there wasn't - there weren't many clients that were open about their perinatal disorder, or even ready or willing to acknowledge it. And now we're seeing a larger volume that's like this is really hard and it's not just the pandemic, like pregnancy is hard growing is hard transitioning.” OP8
3.A.2 Obstetric professional capacity to facilitate patient's access to mental healthcare	3.A.2i. Limited contact with colleagues restricted opportunities, both formal and informal, to develop strategies to improve delivery of mental healthcare.	“Some things just feel less familiar procedurally like, and I feel like because we're more isolated our, our discussions are less robust and so I feel like my clinical knowledge is harder to keep up because it's just we're not working in the same way.” OP1
	3.A.2ii. Limited obstetric professional time to assess or properly assess and refer patients to mental healthcare.	“I think mostly in that we have so many more demands on our time, uh, that we have less time when we identify someone with a positive screen to then assess them and, uh, come up with a treatment plan together.” OP1
3.B Availability Staffing and other resources needed to support the healthcare system capacity to respond to patient mental healthcare needs.		
3.B.1 Staffing required to respond to increase in mental healthcare needs	3.B.1i. Increases in the volume of patients needing mental health appointments during COVID-19.	“I found that getting access to therapists and getting access to mental health providers was more challenging with the pandemic, just because there was a greater need.” OP10
	3.B.1ii. Shortages in supply of perinatal mental health professionals during the COVID-19 pandemic resulted in reduced availability for appointments.	“It's gotten incredibly hard to get people into therapy. Supply and demand is just - there's more demand than supply... It's really, um, you know, these people are flooded with folks and there's just not enough providers around.” OP6
3.B.2 Resources required to respond to patient mental healthcare needs such as infrastructure	3.B.2i. Preferred mode of treatment impacted mental healthcare professional availability during COVID- 19	“There aren't a lot of s who have face to face time and virtual visits don't work for everyone and telephone calls don't work for everyone.” OP8
	3.B.2ii. Loss of access to treatments, classes, and support groups during COVID-19	“A lot of the group therapies now, of course, were canceled, so, you know, they didn't have that resource, particularly in my substance use where they, you know, they do a lot of support from their group and, you know, they didn't have that.” OP5
3.C Affordability Direct, indirect, or opportunity costs of accessing the healthcare system, the extent of reimbursement for care provided and the services sought, and treatment recommendations influenced by these considerations.		
3.C.1 Direct costs to patients for mental healthcare	3.C.1i. Costs associated with out-of-pocket expenditures for mental healthcare and medications were especially challenging to address for those who lost health insurance during COVID-19 pandemic.	…probably a realistic barrier is just financially,' cause a lot of women did lose their jobs or their spouses lost their jobs, which they might have lost insurance. And so if you are referring them to some therapists, you know, those costs more money than most people want to or can't afford, especially without insurance. And that could have prevented people from seeing someone as well as medication. OP4
3.C.2 Indirect costs to patients for mental healthcare	3.C.2i. Increase in telehealth reduced the indirect costs for participation by (1) reducing cost of childcare and transportation to care.	“So I think it [Telehealth] is helpful for people who have lack of transportation or have a lot of kids already or responsibilities they couldn't physically take time off from the responsibilities to go to an appointment.” OP4
	3.C.2ii. Increase in telehealth introduced new indirect costs to access mental healthcare including access to a cell phone, the necessary minutes, and network coverage.	“Well a lot of times our patients didn't always have internet access and you were just talking to them over the phone or they had poor internet access. A lot of my patients' kind of, you know, have financial challenges and stuff and they wouldn't have minutes left on their phones or, you know, things like that.” OP5
3.C.3 Opportunity costs to patients for mental healthcare	3.C.3i. Increase in telehealth allowed for decrease in time commitment to access mental healthcare.	“The idea of coming into the office to talk to a provider about mental health meds that they're not sure they want to get on anyway, for example. I think the idea of, oh, we can just schedule a minute telehealth chat, you know, they're like, all right, fine, I'm open to that. Right. That feels like a reasonable amount of effort to put into this, especially if they're really depressed. So, I think that's sort of worked on both sides. [name], our prescriber, has a little bit more room in her schedule for those visits and it's a little easier for folks to opt in. OP7
3.D Accessibility Patient resources needed to access in-person or telehealth mental healthcare.		
3.D.1 Supply-side changes in mental healthcare delivery and mental health resources required	3.D.1i. Increased reliance on telehealth reduced burdens to accessing care related to childcare	“There's a lot of, you know, missed visits. I think a lot of that's related to childcare and, you know, the schools being inconsistent.” OP5
	3.D.1ii. Increased reliance on telehealth to reduce transportation barrier	“Patients who are going to show for their visit in person, we're going to show for their zoom visit and it honestly, maybe we were able to capture more patients via zoom because we didn't have the transportation barrier. We did have the internet barrier but not the transportation barrier.” OP12
	3.D.1iii. Increased reliance on telehealth required new resources to access mental healthcare, including privacy, available minutes, and internet access.	Ensuring that you had their attention a lot of times they have their children with them or they're not in a private setting, so I don't think they're answering the questions honestly,' cause, you know, they may be sitting in the room with their boyfriend or husband or friends. Had one patient who was using the internet at the library OP5
	3.D.1iv. Alterations to the technologies and skills required to facilitate telehealth.	“So we already use tech translators, we use iPad and there's a way to kind of like bring your speakerphone into a zoom conversation, but it's a nightmare. I mean, really, it's like I'm 50 years old. It's a little bit of technical nightmare for me. I went back to grad school for anyone. Yeah, but I feel like I'm a little bit more – yeah, but I mean I feel like that can be a barrier, but we do joke.” OP13
3.D.2 Demand-side changes, specifically in the resources needed by the patient to access appointments.	3.D.2i. Increased difficulty obtaining a reliable or private mode transportation for in-person appointments	“A lot of my patients don't have their own transportation, so it was not wanting to come in because they'd have to take the bus and be exposed that way.” OP2
	3.D.2ii. Increased difficulty obtaining reliable childcare for in-person visits due to closing schools and loss of social network	“There's a lot of, you know, missed visits. I think a lot of that's related to childcare and, you know, the schools being inconsistent.” OP5
3.E Accommodation Extent to which healthcare systems and policies are organized in ways to address the constraints experienced by and needs of the patient.		
3.E.1 COVID-19 pandemic altered the ability for healthcare systems address patient needs.	3.E.1i. Delay between a mental health screen being administered and discussion with a mental healthcare professional.	“When we gave them the questionnaire and let them fill it out in the privacy of the room while they're waiting on the doctor. It just seemed like you could address the issues right there. You know, [during the pandemic], we'd have patients that we would ask to follow up for a phone visit. They didn't, they didn't always make, um, their visits a priority.” OP5
	3.E.1ii. Loss of in person supports such as translators	“The language barriers were hard… calling somebody wasn't always easy. You had to figure out accommodations for a translator and that just kind of slowed down the process or made the, you know, you did the best you could, but sometimes you were talking to an 11-year-old about their mom.” OP6
3.E.2 COVID-19 pandemic altered the ability for policies to address patient needs.	3.E.2i. Ongoing policy restrictions limiting parity and flexibility for the “virtual visit,” such as lack of reimbursement when patients participate by phone and not Zoom.	I mean, really, it's like I'm 50 years old. It's a little bit of technical nightmare for me. I went back to grad school for anyone. Yeah, but I feel like I'm a little bit more - yeah, but I mean I feel like that can be a barrier, but we do joke. And I remember that my frustrations during is like, you know, the patient can't get on Zoom and then you have to switch it to a telephone visit and then we're not going to get paid for that telephone visit, which isn't the motivation, but we have to keep the lights on or, you know, the patient lives literally. We have patients who live where there's almost no cell service and they're out in their backyard. OP13
	3.E.2ii. Introduction of new constraints and potential barriers to care due to organizational policies initiated to mitigate risks associated with COVID-19.	“It's just a general sense that, you know, we get, that some patients have, are just not coming in for care or don't feel welcome because we have mask policies now, you know, for the hospital, patients have to show proof of vaccination or a recent negative test. So I feel like that is a health system barrier that may be, might be limiting some patients coming in to see us.” OP1
	3.E.iii. Changes in visitation policies	“I think it's really hard for folks to access things like childcare for their visits, so that was a real barrier. And when we went back to, to primarily in office visits, that was a big part of our discussion, was are we comfortable having kids in the building now?”OP7
3.F Acceptability Development of patient or obstetric professional perception of the receipt of mental healthcare, including encounters with (administrative) and clinical staff.		
3.F.1 Patient or obstetric professionals' perception of quality of available mental healthcare received	3.F.1i. Impact on quality of care received due to perinatal care professional burnout	“We're stressed, we're exhausted, we're tired, we're constantly wearing K95s, you know, or we're not doing that and putting our health at risk. And so there's this balance to be found. So I think providers, the OBs and midwives, the default is to give them a script and refer them out, because there's nothing else they can do because they [providers] just don't have the capacity.” OP11
	3.F.1ii. Impact of COVID-19 on mental healthcare delivery on continuity of care.	I also have a pretty robust patient panel, so patients were having to get shifted to other providers if I wasn't available. And then that kind of created a little bit of a disconnect with some of my patients or just they were sad, like they wanted to see who they wanted to see, but also if I'm not available, I'm not available. So that kind of happened to a couple providers as well…. But in general, there was limited availability and so I think that that was hard on the patients and hard on us as well. OP2
3.F.2 Alterations in perception of quality of relationship with clinical team.	3.F.2i. COVID-19 risk mitigation strategies decreasing the quality of the patient-obstetric professional relationship.	Um, I feel like the other things that have, um, become a problem for me or, or how it's impacted, how we deliver care. Is that even just something as simple as wearing a mask I think gets in the way of us getting to know each other better. I Feel like I don't know my patients as well because we're wearing a mask. You don't see those facial expressions, you don't - we don't touch as much as we use too, so that's another thing people feel like it's not okay to, to make contact unless it's for a very specific reason. OP1
	3.F.2ii. Telehealth decreasing perception of patient-obstetric professional trust.	“I think specifically our lower income community is our communities of color. There's a lot of provider mistrust that comes into play too…I think Telehealth takes a lot of the trust out of. It's hard to trust somebody on the other end of the screen.” OP8

## Discussion

Public health crises increase the risk of patients developing mood and anxiety disorders and impact obstetric professionals’ ability to address these mental health conditions (45). The challenges of accessing treatment for mood and anxiety disorders are further exacerbated for perinatal women who are economically marginalized (36). This study developed a six dimensional taxonomy of perinatal mental healthcare access among economically marginalized perinatal women during a public health crisis. Within each dimension, we identified specific components and influential factors that shaped access to care. By examining access to care in a system under the strain of a global public health crisis, our analyses demonstrate how the dimensions of perinatal mental healthcare access were dynamically and distinctly impacted by a series of sudden transformations across the social, political, and organizational context in which perinatal mental healthcare is delivered. Our findings suggest that each dimension of accessing perinatal mental healthcare was impacted and offers new insights into how factors within each dimension of access influences perinatal mental healthcare. We first identified specific components of each dimension of access among economically marginalized perinatal women. We then identified influential factors that can inform potential opportunities to mitigate access barriers through policies and programmatic interventions.

We identified dimensions generally consistent with the “5 As of Access” first developed by Penchansky and Thomas in the 1980s. The primary motivation for Penchansky and Thomas (1981) to develop the As of Access was to develop a more precise definition of access and pathways to increased satisfaction for healthcare consumers (31). The focus of the framework was the fit between the patients’ needs and the healthcare system’s ability to meet those needs (31). In our analysis, we developed a taxonomy integrated with the 6As framework describing dimensions that detail a pathway to mental healthcare where healthcare systems are at the center of facilitating the process. Our findings position the expanded 5A’s of Access by Levesque et al., 2013 as a significant contribution to the field of perinatal mental health, demonstrating the significance of the approachability. While our findings align with existing 5A’s by Levesque et al., 2013 (32) literature, this study extends these findings by highlighting how public health crises introduce unique factors for perinatal women within these dimensions, such as the technological affordability’ of telehealth minutes within the dimension of affordability.

First, the healthcare system has to create opportunities and facilitate a patient’s ability to contemplate and prepare for mental healthcare (Approachability). The healthcare system then requires the staffing and resources needed to support the patient to meet any identified mental healthcare needs (Availability) and is able to offer services in ways that do not preclude patient receipt due to associated direct or indirect costs (Affordability). The healthcare system then is charged to assess the resources a patient needs to access the mental healthcare appointment (Accessibility) and if there are any barriers to access, be able to address those barriers (Accommodation). Finally, the healthcare system needs systems to identify and respond to any poor perceptions that arise during the receipt of mental healthcare (Acceptability). We found that the dimensions of the pathway identified from data from of obstetric professional who serve economically marginalized perinatal women, converged with the perspectives of economically marginalized perinatal women seeking mental healthcare during the COVID-19 public health crisis.

Notably, our use of a sixth domain of access is consistent with subsequent formulations of the As of Access Framework developed by others who argued that the sixth A, “approachability,”—an women’ ability to contemplate and prepare for healthcare—is integral to access and should be applied whenever using the framework (32). Our analyses suggest that “approachability” is particularly important for mental health conditions among populations that are economically and racially marginalized. In the context of access dimensions, approachability is heavily influenced by a patient’s perception of safety. Women from these communities’ experience challenges within healthcare settings due to longstanding and reoccurring economic and racial injustices that occur at both the collective and individual levels (46). Illustrative of how healthcare systems themselves may impede approachability, one respondent in our sample spoke to having not disclosed concerns of a perinatal mental health conditions because of it potentially leading to a second psychiatric hospitalization. This sentiment reflects study findings among economically and racially marginalized women on mental health related experiences in healthcare settings. For example, Black women are more likely than any other racial/ethnic group to be physically restrained (47). Minoritized and marginalized women are more likely to be hospitalized for psychiatric conditions than higher income and non-minoritized women who report the same symptoms. Efforts to address mental health related experiences among economically marginalized and minoritized women remains a critical undertaking of our healthcare systems (46). Perinatal women and obstetric professionals both identified the limited capacity of healthcare systems to address mental health conditions given the many strains on the healthcare system amidst the pandemic. Despite these barriers, professionals did report that the larger conversation on mental health during the pandemic did help to normalize speaking about mental health during their clinical visits.

## Limitations

While the dimensions and components identified in the study might be universal, the taxonomy is most transferable to economically marginalized populations. Self-selection into the study may have resulted in a sample of obstetric professionals uniquely invested in addressing perinatal mental healthcare disparities and may have resulted in a more expansive set of themes than less invested professionals. In addition, the obstetric professionals who participated in the study were homogeneous on several sociodemographic factors (e.g., gender, race, ethnicity) while perinatal women were far more heterogenous. The lack of diversity among obstetric professionals in our sample is consistent with studies that demonstrate underrepresentation of Black and Latinx women in the obstetric workforce (48). These differences in racial, ethnic, and economic situation of obstetric professionals and the perinatal women interviewed can introduce blind spots in the perception of access to perinatal mental healthcare amidst a public health crisis. Therefore, further work is needed to extend and assess generalizability of findings across diverse samples of obstetric professionals. Our dimensions of access arose from obstetric professionals and perinatal women amidst a public health crisis (COVID-19 pandemic) which caused a shift in healthcare delivery (e.g., increase in telehealth). However, to advance health equity, identification of the strategies to address these changes in access to mental healthcare should center perinatal women who are economically marginalized. Finally, our sample exclusively captured the experiences of individuals who identify as female. Consequently, our findings may not generalize to transgender, non-binary, or gender-expansive individuals navigating the perinatal period. Future research should include perinatal individuals of diverse gender identities to investigate the perinatal experiences across the full spectrum of gender identities.

## Implications for policy and programmatic innovation

One goal of our study was to identify potential ways to strengthen our mental healthcare system and mitigate the potential impact of a future public health crisis on perinatal mental healthcare access, particularly for economically marginalized perinatal women. More specifically, the factors identified to address the six dimensions of access provide descriptions of activities that can be targeted for clinical and/or policy intervention to both increase and advance equitable mental health access. Potential policy or programmatic interventions may assist in mitigating the impact of a public health crisis on access to care. However, our research suggests that the strategies required benefit from a clear and in-depth understanding of the specific dimensions of access of care targeted. To illustrate this, we provide a summary of the policy or programmatic interventions that might be considered in response to each dimension of access to care:

### Approachability

Partnerships and investments in community-based spiritual, health, and social systems and networks are an integral part of addressing the factors associated with approachability (49). For example, our Advisory Council members emphasized that doulas and midwives are critical partners in facilitating the contemplation and preparation required for accessing mental healthcare. These perinatal professions provide longstanding established birthing care approaches and engage practitioners that more commonly arrive, themselves, from the communities they serve. Additionally, opportunities exist for investments in local organizations that are also led by the communities that they serve. Prior calls to the field suggest such investments need to expand the parameters for funding, specifically facilitating support for organizational infrastructure, capacity building, and community-driven priority setting and achievement (49). Supports are also needed to empower local community-based organizations to work with healthcare systems to facilitate contemplation and preparation for our accessing mental healthcare during the perinatal period.

### Availability

The COVID-19 pandemic increased prevalence rates of perinatal mental health conditions creating additional demand for perinatal mental healthcare. At the same time, perinatal mental healthcare systems available to address this additional demand confronted additional stressors amidst the public health crisis (50, 51). Opportunities exist to strengthen the capacity of the perinatal workforce to screen, assess, treat, and/or refer patients with perinatal mental health disorders amidst a public health crisis. Efforts, like the Perinatal Psychiatry Access Program model, aim to strengthen the capacity of the perinatal workforce to provide mental healthcare (52). Such efforts might also provide additional supports that are responsive to the transformations in perinatal care during a public health crisis. For example, Perinatal Psychiatry Access Programs or other models of integrated care might provide trainings on how to integrate perinatal mental healthcare into telehealth appointments or to facilitate continuity of care when implementing precautions regarding infections. Recent federal policy efforts, including the Consolidated Appropriations Act passed in December 2022 (53), provide longer-term solutions by including new general psychiatry residency positions, funding and developing more fellowships in maternal and reproductive mental health, providing requirements for improved Medicaid provider directories, and creating new funds to support diversifying the workforce, including peer support providers. These efforts could help build the workforce available to provide care in response to the demand that emerges during a public health crisis. Additionally, efforts to increase rates for mental healthcare, extend workforce, and incentivize participation in the Medicaid program are also important initiatives to address availability for economically marginalized women (54).

### Affordability

The pandemic placed additional financial strain on perinatal women and their families. Not surprisingly, respondents, who were already economically marginalized, reported additional sensitivities to the direct, indirect, and opportunity costs associated with perinatal mental healthcare. Solutions to addressing the affordability of care include strategies that reduce the direct costs associated with perinatal mental healthcare, such as affordable premiums for low-deductible plans and improving plans by reducing deductibles and other cost sharing charges in public and marketplace plans. Strategies might also include extending the out-of-pocket maximum structure of the Affordable Care Act and replacing the current annual cap on out-of-pocket costs with monthly caps on out-of-pocket costs. The monthly out-of-pocket monthly caps would align more closely with how women are paid at jobs and manage household budgets while reducing the potential for cost-sharing peaks due to hospital or emergency intervention (55). New indirect costs are also associated with accessing telehealth. Efforts to address the telehealth technology barriers, including internet services, are critical part of addressing affordability of these new innovations. Opportunities also exist to extend programs that reduce these indirect costs, like the Telehealth Network Grant Program (56), that invested in telehealth technologies for nonprofit entities providing services to rural and medically underserved directly. Finally, efforts to mitigate the impact on both indirect and opportunity costs might include investments in childcare programs and the social drivers of health and healthcare.

### Accessibility

Challenges in ensuring the required resources to provide timely receipt of perinatal mental healthcare may benefit from innovative finance models. For example, value-based programs reward health care providers with incentive payments for access to quality care. These types of programs are supported by the Centers for Medicare & Medicaid Services and a part of CMSs strategy to reform how health care is delivered and paid for (57). New York State’s Medicaid Maternity Care Value Based Payment (VBP) Arrangement was designed to allow maternity care providers to focus on integrated prenatal, delivery, postpartum and newborn care. Quality measures related to each of these stages of care reinforce the care connections built into the Arrangement and provide standardized measure for maternity care providers statewide. Value-based payment models offer promise in facilitating the continuity of care that is optimal to ensure mental healthcare is accessible throughout the perinatal period (58). Despite the promise of these resources and models, widespread understanding and implementation still faces challenges. Reimbursement for mental health services in obstetric settings is an ongoing challenge. Obstetrics care is typically reimbursed with a global payment procedure code encompassing all prenatal, intrapartum, and postpartum care, from the first visit after the confirmation of pregnancy to the final postpartum visit. There are lessons to be learned from other countries’ payment, policy, and care models. Other countries, for example, take an individualized approach to global payments, allowing for payment adjustments related to risk and differential intensity of prenatal, intrapartum, and postpartum care. A differential payment structure that provides risk-adjusted compensation for the additional resources required to address perinatal mental health conditions, could facilitate improved integration of mental health care into obstetric practice. Another barrier to access is the lower rate of reimbursement for mental healthcare when compared to any other type of medical care, such that many mental healthcare professionals only take cash (59).

### Accommodation

Perinatal women experience barriers to the accommodation of their needs related to accessing mental healthcare such as childcare during visits. To address these barriers, health care settings should develop a formal process for screening for unmet social needs and access during visits and make referral to services to meet those social needs (60). The US Department of Health and Human Services have made a call for and efforts to address unmet social needs. Efforts include the development of backbone organizations focused on aligning health and social care through the creation of a community care hub. Hubs leverage community capacity and expertise to allow for efficient and scalable approaches health and social care services (61). State Medicaid programs have historically had relatively limited flexibility in addressing the social drivers of health. However, recent innovations have led to calls for policymakers to look to the Medicaid program to improve this (62). First, certain non-clinical services continue to be available to home and community-based service programs to support women with disabilities and the elderly (63). More recently, a range of state plans and waiver authorities (e.g., Section 115, 1905(a), 1915(c)) are available to support services including employment and housing support, as well as case management and peer support services (62).

### Acceptability

The development of rapport is an important component to addressing factors associated with acceptable mental healthcare for perinatal women. Acceptability is related to the perception of the receipt of care, and the development of rapport is important to patient experiences with care. Rapport is defined as a ‘harmonious relationship’ and relates to collaboration and parity between patient and physician (64). Effective rapport has been shown to improve patient compliance with treatment, clinical outcomes and patient satisfaction (64). Rapport is established early (e.g., questions asked) and must be continually cultivated by clinic staff and the clinical team (e.g., commitment to maintain the perinatal women mental and physical health). To address barriers to developing rapport various approaches have been developed. Approaches include continued education demonstrating how rapport is developed and sustained (65) and resources dedicated to building rapport across care delivery platforms such as Embodied Conversational Agent Technology. Moreover, efforts to support patient-centered care should continue. Patients are partners with health professionals and health professionals should not only consider the clinical perspectives but mental, social and financial perspectives as well (66). Even at the clinic level, human resource practices, organizational culture and physical environment have been shown to have effects on quality rapport and should be considered when addressing acceptability (67). Additionally, opportunities exist for policies that seek to improve patient-centered care that incentivizes process for shared decision-making, such as reconfiguring protocols for training and educating healthcare professionals to center the patient experience, identifying provider reimbursement models that incentivize and reward patient-centered medical homes and share decision-making, and ensuring that accreditation requirements set clear expectation for patient-centered care and shared decision-making. Finally, the social conditions that economically and racially marginalized perinatal women experience can make them more vulnerable to trauma. Trauma-informed care (TIC) is an approach that encourages awareness of needs of patients, clinicians, and staff who may have experienced trauma (68).

Our findings suggest healthcare systems need to develop plans for accessing care during various public health crises, including those related to infectious disease and natural disasters where in-person appointments might not be feasible. Developing plans for perinatal professionals and patients on the use of distance-based treatment modalities will be important tools for emergency preparedness. While the dimensions identified were among data collected during and specific to the COVID-19 pandemic, the dimensions have implications for post-pandemic healthcare settings. Given the stressors related to the pandemic, we found an increased knowledge of and need for mental healthcare. Perinatal professionals considered means to address barriers beyond individual patient behaviors such as the provision of childcare. Moreover, there was an increase in the use of telehealth that while increased access, introduced new barriers such as costs related to technology needed to provide and receive service using that technology.

## Implications for future research

In addition to the implications for policy and practice, our study also has implications for future research. First, opportunities exist to develop surveys that facilitate an understanding of whether findings reported in this article are generalizable across the limited sample interviewed. Such understandings could be critical to setting a stage for identifying strategies that are responsive to the challenges confronting perinatal mental healthcare access and strengthening the system in advance of any future public health crises. Moreover, our findings also suggest that factors influencing perinatal mental healthcare access amidst a public health crisis are amendable to policy and programmatic intervention. We outline the conclusions of our community partners and research team on the implications of these findings for policy and programmatic intervention below.

## Conclusions

Perinatal mental health is a core aspect of overall societal health. Integrating care for perinatal mental health into obstetric care, providing needed resources, and ensuring the appropriate financial incentives, policies, and implementation models are available to do so, carries the potential to reduce preventable maternal mortality and improve the overall health of mothers and families. We have an opportunity to optimally impact the health of our nation’s mothers, children, and families by improving perinatal mental health and reducing maternal mortality.

Our study reflects changes in attitudes towards mental health and mental health treatment and shifts in provision of healthcare that occurred during the COVID-19 pandemic. We developed a taxonomy comprising six dimensions of perinatal mental healthcare access responding to those changes and shifts with a description of factors and opportunities for intervention within each dimension of our taxonomy. The opportunities described can contribute to policymakers and obstetric professional’s ability to anticipate and respond to challenges to perinatal mental healthcare access during public health crises and the post COVID-19 healthcare environment. These types of efforts provide important implications for efforts to advance perinatal mental healthcare equity.

## Data Availability

The datasets presented in this article are not readily available to protect participant confidentiality. Requests to access the datasets should be directed to Thomas Mackie, tmackie@umass.edu.

## Appendix 1 Semi-Structured Interview Guide For Prescribing Perinatal Providers

### Section 1: Background & Pandemic Impact

1. What types of care do you provide and what training do you have?
2. Describe your experience before and during the pandemic.
3. Describe the community you serve.

### Section 2: Detection

4. Describe your screening approach before the pandemic.
5. How has the pandemic changed screening?
6. What facilitated screening?
7. What challenges did you face?

### Section 3: Assessment/Diagnosis

8. Has the pandemic affected response to positive screens?
9. Has it affected your ability to assess?
10. What facilitated assessment?
11. What challenges did you face?

### Section 4: Triage

12. Describe triage approach before the pandemic.
13. Has the pandemic changed triage?
14. Did access change for certain communities?
15. What facilitated triage?
16. What challenges did you face?

### Section 5: Treatment

17. How do you initiate treatment?
18. Has the pandemic changed treatment?
19. Has it affected referrals/resources?
20. Has it affected adherence?
21. What facilitated treatment?
22. What challenges did you face?

### Section 6: Treatment Follow-Up

23. How do you follow up with patients?
24. How has the pandemic changed follow-up?
25. What facilitated success?
26. What challenges did you face?

### Section 7: Continuity of Care

27. How do you ensure ongoing care after postpartum visit?
28. Has the pandemic changed this process?

### Section 8: Engagement and Trust

29. How do you connect with patients?
30. Has the pandemic affected connection?

### Section 9: Health Equity

31. What does health equity mean to you?
32. What changes have you made to promote equity?
33. Do you use an antiracist approach?
34. How has the pandemic affected equity?

### Section 10: Provider Well-Being

35. Did you experience burnout or challenges?

### Section 11: Access Programs

36. Have you worked with access programs?
37. Have you used them for minority communities?

## Appendix 2 Semi-Structured Interview Guide For Perinatal Women

### Section 1: Background

38. Can you tell us about yourself and your pregnancy/postpartum experience?
39. What type of care did you receive?
40. Describe your community and support system.

### Section 2: Experiences During the Pandemic

41. How did the pandemic affect your pregnancy or postpartum experience?
42. Did it change how you accessed care?
43. What barriers or supports did you experience?

### Section 3: Mental Health and Screening

44. Were you ever asked about your mental health during pregnancy or postpartum?
45. How did providers screen or check in with you?
46. Did the pandemic change this experience?

### Section 4: Diagnosis and Support

47. Were you ever told you might have a mental health concern?
48. What happened after that?
49. Did you feel supported in understanding your mental health?

### Section 5: Treatment and Care

50. Did you receive any treatment (therapy, medication, support groups)?
51. How did you access these services?
52. Did the pandemic affect your ability to receive treatment?

### Section 6: Follow-Up and Continuity

53. Did providers follow up with you about your mental health?
54. What happened after your postpartum visits ended?
55. Did you feel continuity of care?

### Section 7: Connection and Trust

56. Did you feel comfortable sharing concerns with providers?
57. Did the pandemic affect your relationship with providers?

### Section 8: Health Equity

58. Do you feel your identity (race, ethnicity, insurance, gender identity) affected your care?
59. What barriers or inequities did you experience?
60. What would improve care for people like you?

### Section 9: Closing

61. Is there anything else you would like to share about your experience?
62. Would you be willing to participate in a follow-up interview?

## Appendix 3 Composition of Advisory Councils

**Table:**

Advisory Council	Composition	Number of Members
National Advisory Board	Representatives from national advocacy and service organizations, professional societies, and the research community.	13
Massachusetts State Advisory Board	At least one representative from: (1) women with lived experience, (2) perinatal care professionals, (3) community-based programs, (4) health plans, and (5) state policymakers.	7
Washington State Advisory Board	State-level advisory board (composition varies by state).	10
New Jersey State Advisory Board	State-level advisory board (composition varies by state).	13
PSI Best Practice Committee for Perinatal Mental Health Equity	Includes midwives, peer supporters, doulas, social workers, counselors, lactation supporters, and obstetric clinicians serving underserved populations.	12
Lifeline for Moms Women with Lived Expertise Advisory Board	National advisory council of women with lived experience of perinatal mental health challenges from marginalized communities.	20
